# Aiming for small is a crime

**DOI:** 10.4103/0019-5049.60486

**Published:** 2010

**Authors:** SS Harsoor, D Devikarani

**Affiliations:** Editor, Indian Journal of Anaesthesia; 1 Associate Editor, Indian Journal of Anaesthesia, No. 21, 2^nd^ Cross, Kirloskar Colony, Basaveshwar Nagar, 2^nd^ Stage, Bangalore – 560 079, India. E-mail: editor@ijaweb.org

With immense pleasure, I thank all the members of Indian Society of Anaesthesiologists (ISA) for having bestowed confidence in me to lead our esteemed journal – Indian Journal of Anaesthesia (IJA).

Ever since its first publication in 1953, the journal has seen several ups and downs in its long journey until 1981. From then onwards, there is no looking back, and the journal has been constantly making an upward journey as regards the quality of its content, regularity and popularity, with its circulation crossing the first 5 digits number in the year 2010. The very survival of any scientific medical journal is dependent upon the quality of its presentations and the validity and reliability of the reviews published by the journal.[1] The ultimate goal is to translate the sound and scientific research into anaesthetic practice and thereby improvising the health care practices for the benefit of the community.

The impact factor that has been defined as the ‘frequency with which the average article in a journal has been cited in a particular year’ is the most common bibliometric quantitative parameter or criterion in use today[2] and has mostly replaced subjective criteria used in the past to determine the journal quality and prestige. It is a dynamic parameter[3] and an indicator of the editorial quality of a journal. It is our constant endeavour to make IJA as one of the most cited journals and quality contribution from each and every member of ISA will be the deciding factor. With the active participation of all the members of ISA, there can be a sea change in the journal and IJA can change its face and contribute to the world anaesthesia literature in a significant way.

The famous editor and author Richard Smith expresses his serious concern that medical journals have become “creatures of drug industry”.[4] It is the time, we move above all these concerns, and continue with unbiased and stringent peer review process to make all the articles acceptable to specialty at large.

I take this opportunity to thank all the previous editorial board members and the team of peer reviewers who have rendered their honest services to help the journal to progress and also to keep the knowledge tree growing. At the same time, I earnestly appeal to all the new authors, peer reviewers and the editorial members to get involved in our esteemed journal and contribute their share of knowledge and expertise for the upliftment of our journal.

We need a change. This is the right time to ignite the minds of fellow anaesthesiologists. As Dr A. P. J Abdul Kalaam says, “Given the vast resources, aiming for small is a crime”. So “think big and progress”.
